# Towards Clinical Applications of Blood-Borne miRNA Signatures: The Influence of the Anticoagulant EDTA on miRNA Abundance

**DOI:** 10.1371/journal.pone.0143321

**Published:** 2015-11-23

**Authors:** Petra Leidinger, Christina Backes, Stefanie Rheinheimer, Andreas Keller, Eckart Meese

**Affiliations:** 1 Department of Human Genetics, Medical School, Saarland University, Building 60, 66421, Homburg/Saar, Germany; 2 Department of Clinical Bioinformatics, Saarland University, Building E2.1, 66123, Saarbrücken, Germany; University of Connecticut Health Center, UNITED STATES

## Abstract

**Background:**

Circulating microRNAs (miRNAs) from blood are increasingly recognized as biomarker candidates for human diseases. Clinical routine settings frequently include blood sampling in tubes with EDTA as anticoagulant without considering the influence of phlebotomy on the overall miRNA expression pattern. We collected blood samples from six healthy individuals each in an EDTA blood collection tube. Subsequently, the blood was transferred into PAXgene^TM^ tubes at three different time points, i.e. directly (0 min), 10 min, and 2 h after phlebotomy. As control blood was also directly collected in PAXgene^TM^ blood RNA tubes that contain a reagent to directly lyse blood cells and stabilize their content. For all six blood donors at the four conditions (24 samples) we analyzed the abundance of 1,205 miRNAs by human Agilent miRNA V16 microarrays.

**Results:**

While we found generally a homogenous pattern of the miRNA abundance in all 24 samples, the duration of the EDTA treatment appears to influence the miRNA abundance of specific miRNAs. The most significant changes are observed after longer EDTA exposition. Overall, the impact of the different blood sample conditions on the miRNA pattern was substantially lower than intra-individual variations. While samples belonging to one of the six individuals mostly cluster together, there was no comparable clustering for any of the four tested blood sampling conditions. The most affected miRNA was miR-769-3p that was not detected in any of the six PAXgene blood samples, but in all EDTA 2h samples. Accordingly, hsa-miR-769-3p was also the only miRNA that showed a significantly different abundance between the 4 blood sample conditions by an ANOVA analysis (Benjamini-Hochberg adjusted p-value of 0.003). Validation by qRT-PCR confirmed this finding.

**Conclusion:**

The pattern of blood-borne miRNA abundance is rather homogenous between the four tested blood sample conditions of six blood donors. There was a clustering between the miRNA profiles that belong to a specific blood donor, but not between any of the four tested blood sampling conditions. The results show a limited overall impact of the blood sampling conditions on the miRNA pattern. Notwithstanding, the abundance of single miRNAs can be significantly altered by different blood sampling conditions.

## Background

Among the most studied nucleic acids are small non-coding RNAs. Currently, release 21 (June 2014) of the miRBase [1, 2] contains 28,645 entries representing hairpin precursor miRNAs, expressing 35,828 mature miRNA products in 223 species. For *Homo sapiens*, 2,588 different mature miRNAs are currently included in this database. The small non-coding miRNAs are known to be involved in crucial biological processes such as proliferation, apoptosis, differentiation, or development [3–5]. More than 50% of all genes in the human genome are known to be miRNA targets and thus, miRNAs are involved in the regulation of a manifold of metabolic and regulatory pathways. Others and we have analyzed the potential of blood-borne miRNAs as non-invasive disease biomarkers. In our previous studies we have shown diseases-specific miRNA expression profiles in whole peripheral blood [6].

It is known that many of the nucleated cells in blood rapidly respond to changes in their environment, and as a result, show changes in gene expression almost immediately after phlebotomy reflecting environmental changes like hypothermia, hypoxia, stress, and contact with foreign surfaces [7]. This has been shown especially for cytokines, chemokines and transcription factors [8, 9]. It has further been shown that anticoagulants can also cause *ex vivo* changes in cytokine production [10–12]. The optimal approach for RNA purification from whole blood is therefore to collect fresh blood in tubes, at the best without anticoagulants, and process the sample as quickly as possible. However, in multicenter studies a delay in RNA isolation up to 24h is a typical situation, and therefore it is necessary to use blood tubes with anticoagulants [13]. But this might lead to *ex vivo* post-phlebotomic expression changes that can cause biased gene expression results. If the blood can not be processed directly after blood withdrawal, rapid cell lysis and appropriate storage conditions of the blood samples should be aspired to obtain accurate results in gene expression analyses. This can be easily achieved by the use of special blood tubes, that contain a stabilizing reagent that directly lyses the blood cells, inhibits *ex vivo* transcription, inactivates cellular RNases and selectively precipitates RNA, like the PAXgene^TM^ blood RNA tubes (Becton Dickinson) or alternatively the Tempus blood RNA tubes (Invitrogen) [8, 9, 14, 15]. All our previous studies mentioned above on miRNA expression pattern in whole blood were based on blood that was stabilized in PAXgene^TM^ blood RNA tubes prior to RNA isolation, to avoid *ex vivo* changes and to ensure standardized conditions for all blood samples. However, in some cases the use of these special RNA-stabilizing blood collection tubes is not possible any more (e.g. retrospective studies) or intact cells are necessary (e.g. approaches that require a pre-treatment of intact cells). For some retrospective studies only frozen whole blood (e.g. in EDTA or Li-Heparin blood tubes) might be available. During the freezing process a large fraction of cells is destroyed and the intracellular RNA will be exposed to RNA degrading enzymes. For studies that require a pretreatment, e.g., a stimulation of intact blood cells, PAXgene or Tempus tubes can also not be used as blood cells are directly lysed after phlebotomy into these tubes. Interestingly, some studies have shown that it might be possible to rescue blood samples collected in, e.g., EDTA tubes and to recover RNA in acceptable quality for gene expression analysis by transferring the whole blood into PAXgene blood RNA tubes [7, 16, 17]. In a recent study, Kim et al [18] compared three different methods to obtain high-quality RNA from frozen EDTA blood including the PAXgene system. It was shown, that transfer of EDTA blood, that was frozen for over 5 years, into PAXgene tubes delivered high-quality RNA in terms of purity (mean A_260_/_280_ ratio of 2.0 ± 0.1) and acceptable integrity (mean RIN value 6.0 ± 1.1). These results are in concordance to our observation. However, Ct values of housekeeping genes differed significantly between the three RNA isolations in the study by Kim et al. A limitation of this study was that they did not compare results of the frozen EDTA blood with fresh EDTA blood or blood that was directly drawn in PAXgene tubes and thus a potential impact of EDTA on the transcriptome remained uninvestigated. Beekman et al. [7] showed that there is a difference in the gene expression pattern of frozen EDTA blood that was transferred after thawing into PAXgene tubes and blood that was drawn in PAXgene tubes directly. However, they did not find a difference between blood that was frozen immediately and blood that was frozen after 3h. Asare et al. [17] found only slight difference in the gene expression pattern (only three deregulated genes) between blood that was drawn in Li-Heparin blood tubes and transferred into PAXgene tubes or blood that was drawn in PAXgene tubes directly. However, Schrauder et al. succeeded in the identification of a breast cancer specific miRNA signature, derived from frozen EDTA blood that was transferred to PAXgene blood RNA tubes after thawing [16]. Nevertheless, to the best of our knowledge there is currently no study on the influence of phlebotomy on the overall miRNA expression pattern.

In the present study we aimed to investigate if blood withdrawal in EDTA tubes and transfer of this EDTA blood (without freezing step) into PAXgene blood RNA tubes has any effect on the overall miRNA expression profile. Further, we analyzed if there is a time dependency in miRNA expression between the time of phlebotomy and the transfer of the blood into PAXgene blood RNA tubes.

## Materials and Methods

### Sample collection

Blood from 6 healthy individuals was collected in one PAXgene^TM^ blood RNA tube (Becton Dickinson, 2.5 ml blood) and one dipotassium EDTA blood tube (EDTA-KE Monovette, Sarstedt, 9 ml blood) per individual. There was no known disease for any of the blood donors. A fixed volume of 2.5 ml blood from the EDTA tube was transferred at three different time points after blood withdrawal (0 min, 10 min, and 2 h) into fresh PAXgene blood RNA tubes to ensure stabilization of the RNA in the blood samples. All PAXgene blood tubes were stored at room temperature until at least 2 h after the last transfer of EDTA blood into PAXgene tubes, to ensure complete lysis of the blood cells, before they were stored at -20°C until RNA isolation.

The participants were between 21 and 55 years old, with a mean age of 36.5. One of the six volunteers was male, the remaining 5 volunteers were female. All participants gave their informed consent. The study was approved by the local ethics committee (Ärztekammer des Saarlandes, 44/05). We obtained written informed consent to participate in the study and to publish the data from all participants.

### RNA isolation

Total RNA of all samples was isolated using the PAXgene miRNA blood Kit (Qiagen) and under standardized conditions using the QIAcube (Qiagen). RNA concentration was measured using NanoDrop-2000 (Thermo Scientific). For RNA quality assessment we used the Bioanalyzer 2100 with Agilent 6000 RNA Pico Chips.

### Microarray analysis

MiRNA expression analysis was carried out using the Agilent microarray platform according to manufacturer`s instructions using SurePrint G3 8x60K miRNA microarrays (Agilent Technologies) that contain 40 replicates of each of the 1,205 miRNAs of miRBase v16. In brief, a total of 100 ng total RNA is processed using the miRNA Complete Labeling and Hyb Kit (Agilent Technologies) to generate fluorescently labeled miRNA. Then the microarrays are loaded and incubated for 20 h at 55°C and 20 rpm. After several washing steps microarrays were scanned with the Agilent Microarray Scanner at 3 microns in double path mode.

The microarray data are freely available in the Gene Expression Omnibus database under Accession number GSE73401.

### Statistical analysis

Using the Agilent Feature Extraction Software we extracted the raw values from the scanned image files. The Total Gene Signal provided by the Agilent Feature Extraction software was used for quantitative data analysis. In parallel, the Feature Extraction Software performs a filtering (gIsGeneDetected) that flags each microRNAs as detected or not detected in all analyzed samples. The result of this procedure was a binary matrix with 24 columns (samples) and 1,205 rows (miRNAs), each entry (i,j) marked by “1” indicated that miRNA i was present in sample j and “0” otherwise. For this matrix a statistical evaluation has been carried out and Venn diagrams as well as Balloonplots have been generated to visualize the distribution of these miRNAs in the four different conditions for the 6 individuals. To test whether the distribution in the different groups was significant for specific miRNAs, permutation tests were done. Here, the group ordering has been randomly perturbed 10^6^ times and the number of random patterns with an at least as extreme distribution as the original one have been counted.

Since the binary matrix just represents a part of all information and does not reflect different abundance of miRNAs we also performed a quantitative analysis using the Total Gene Signal. Normalization between arrays was carried out using quantile normalization. Since low expressed miRNAs potentially add noise, we excluded all miRNAs that have not been detected in at least 25% of all samples. To discover general trends, hierarchical clustering using Euclidian distance was done for miRNAs and samples individually. To compare the patterns per group, analysis of variance (ANOVA) has been carried out and significance values were adjusted for multiple testing by the Benjamini-Hochberg approach. To find miRNAs that are differentially expressed between different conditions, all 6 pair-wise comparisons were done for each miRNA. Since we measured the different conditions for the same individuals, paired t-tests were calculated and significance values were adjusted for multiple testing. Since one main goal of this study was to discover miRNAs that show small intra-individual variations compared to inter-condition variability we calculated for each of the four conditions the coefficient of variation (CV) and compared the average CV to the overall CV.

### qRT-PCR validation

Validation of the expression of the 4 miRNAs that are significantly differentially regulated between 2h EDTA and PAXgene samples using qRT-PCR was performed with the StepOnePlus Real-Time PCR System (Applied Biosystems) and the miScript PCR System (Qiagen) according to manufacturers recommendations. In brief, we used 200ng RNA for conversion into cDNA using the miScript RT II Kit. For PCR 2μl cDNA (1:10 dilution) was used and RNU48 served as endogenous control.

## Results

### Quality and yield of RNA

We collected blood from six healthy individuals each into a PAXgene tube and into an EDTA tube. Blood that was first collected in an EDTA tube was subsequently transferred into PAXgene tubes at three different time points, i.e. directly (0 min), 10 min, and 2 h after phlebotomy. These samples are in the following referred to as EDTA 0min, EDTA 10min, and EDTA 2h, respectively. For the six patients and four data points totaling 24 samples we analyzed the genome wide miRNA expression using human Agilent miRNA V16 microarrays that contain 1,205 mature miRNAs. RNA isolation was done automatically using a robotic workstation for automated purification of nucleic acids or proteins (QIAcube from Qiagen) to reduce potential bias introduced by manual processing. RNA isolation yields average concentrations of 117.85 ± 36.28 in PAXgene samples, 110.05 ± 35.12 in EDTA 0min, 106.90 ± 26.34 in EDTA 10min, and 121.82 ± 44.94 in EDTA 2h samples. The 260/280 absorbance ratios were at 2.05 ± 0.03 indicative for pure RNA without contaminations in all cases. The RNA Integrity Number (RIN) calculated by the software of the Bioanalyzer 2100 was at 7.09 ± 0.38 for the 24 measured samples. Pair-wise hypothesis tests revealed that no significant differences between any of the above quality criteria in any of the four conditions were observed.

### Overall miRNA detection

**Fig 1:**
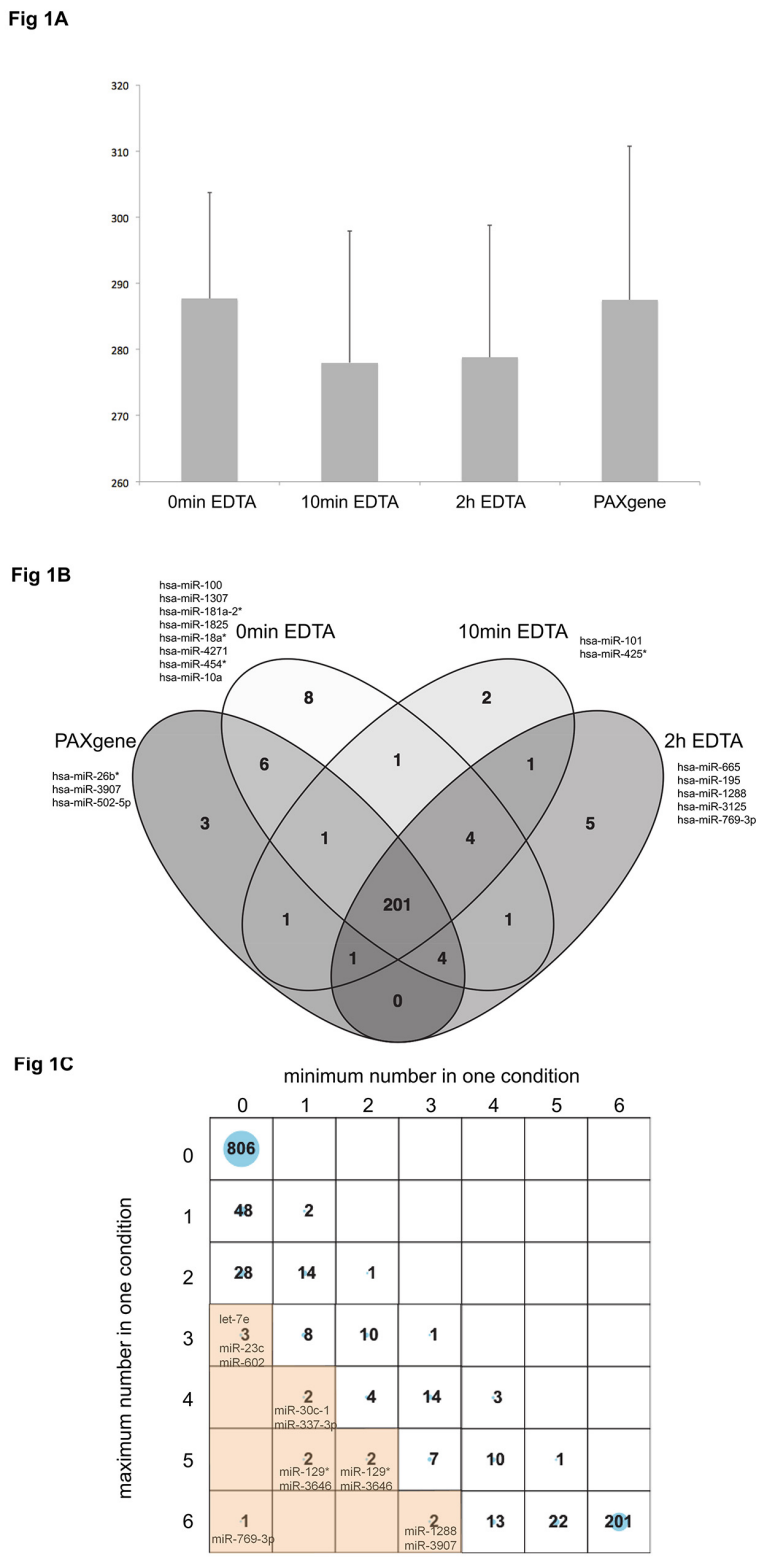
Microarray present call analysis. A) Average number (Y-axis) and standard deviation of miRNA detected in blood samples of six healthy individuals under four different blood sampling conditions (X-axis). Blood was collected in EDTA blood collection tubes and subsequently transferred into PAXgene^TM^ tubes at three different time points, i.e. directly (0 min), 10 min, and 2 h after phlebotomy. As control blood was also directly collected in PAXgene^TM^ blood RNA tubes. B) Venn diagram of miRNAs that were present in all six individuals under different blood sampling conditions. There were 201 miRNAs detected in all individuals under all conditions. No miRNA was detected both in blood that was directly collected in PAXgene^TM^ blood RNA tubes and in blood that was transferred into PAXgene^TM^ tubes 2 h after phlebotomy without also being detected in blood directly transferred or transferred 10 min after phlebotomy. C) Balloon plot of miRNAs that show a difference in frequency under the different blood sampling conditions. The X-axis shows the minimal number of individuals that are positive for a miRNA under one condition. The Y-axis shows the maximum number of individuals that are positive for a miRNA under one condition. The top left balloon denotes the 806 miRNAs that were not detected in any of the 24 samples (minimum and maximum of 0). The lower right balloon denotes the 201 miRNAs that were detected in all samples (minimum and maximum of 6). The orange shaded area presents 12 miRNAs that show a difference in frequency of at least three under one of the four conditions as compared to the other conditions. The largest difference was found for miR-769-3p indicated in the lower left corner that was positive in 6 samples under the 2h EDTA condition and not in any sample of the PAXgene condition.

We initially based our analysis on present calls, i.e., we compared the numbers of miRNAs detected in the blood samples of the six individuals under the four different conditions (PAXgene samples, EDTA 0min, EDTA 10min, and EDTA 2h). Altogether we found 201 miRNAs that were detected in all individuals and under all four conditions. Out of the 1,205 mature miRNAs, 806 miRNAs were not detected in any sample. The remaining 399 miRNAs were detected in at least one sample. On average we observed 288 miRNAs in PAXgene samples, likewise 288 miRNAs in EDTA 0min samples, 278 miRNAs in EDTA 10min samples and 279 miRNAs in EDTA 2h samples. The mean value and standard deviation are presented as bar plot in Fig 1A. Statistical analysis between all pairs of groups did not reveal any significant variation in the quantity of detected miRNAs. A Venn diagram of miRNAs that were present in all six individuals under the different blood sampling conditions is given in (Fig 1B). Overall, we thus found a rather homogenous pattern of the miRNA abundance in all six individuals for each of the four conditions. The complete list of detected miRNAs in the 24 experiments is provided as S1 Table.

### Variation of the miRNA repertoire dependent on the blood sampling

To identify the most variable miRNAs with respect to the present call we calculated for each of them the maximal number of individuals found under one of the four blood sampling conditions and compared it to the minimal number found under one of the remaining conditions. We identified very few miRNAs that were present under one of the conditions but not or less present under one of the other conditions. For example miRNA-769-3p was found in all six individuals under the 2h EDTA condition but not in any individual under the PAXgene condition. One million permutation tests showed in 0.16% of all cases such an extreme distribution (p = 0.0016). Fig 1C specifically indicates all miRNAs with a difference in frequency of at least three samples found under one of the four conditions as compared to the other conditions, i.e. all miRNAs that are at least found in “n” individuals under condition “a” and “n-3” individuals under condition “b”. For example let-7e, miR-23-c and miR-602 are each found in three samples under one condition but not in any sample under any of the other conditions. MiRNA hsa-let-7e was not detected in any individual under the PAXgene blood condition but three times under the EDTA 2h condition. MiRNA hsa-miR-602 was detected in 3 of 6 PAXgene blood samples but not in any EDTA blood sample. As shown in Fig 1C there are only 12 miRNAs that show a difference in frequency of at least three under one of the four conditions as compared to the other conditions.

### Quantitative changes in miRNA abundance

**Fig 2:**
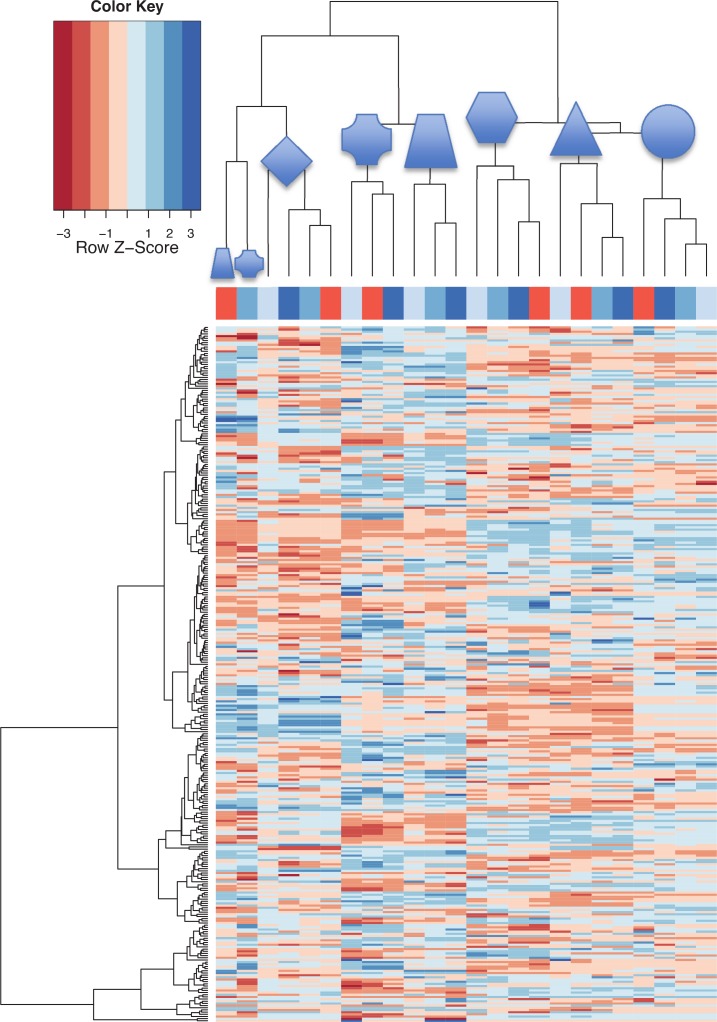
Heatmap of 321 miRNAs detected in at least 25% of samples. The X-axis differentiates between the four different blood sampling conditions. The light blue shading indicates blood that is directly (0 min) transferred into PAXgene^TM^ tubes, the middle blue shading blood that is transferred 10min after phlebotomy and the dark blue shading blood that is transferred after 2 h. Blood that was directly collected in PAXgene^TM^ blood RNA tubes is indicated in orange. The 6 individuals are indicated by symbols on top of the dendrogram. While the four samples belonging to one of the six individuals mostly cluster together there was no comparable clustering for any of the four tested blood sampling conditions.

The above analysis used present calls based on binary data, which allow only differentiation between expressed and not expressed miRNAs. Since the applied microarray technology allows quantification of miRNA expression levels we investigated changes in the abundance of miRNAs in more detail. In the following, we considered miRNAs that were present in at least 25% of all analyzed samples. Data were quantile normalized to account for systematic variations between different microarray experiments. This approach identified 321 miRNAs. For analyzing the overall distribution of miRNA signatures we carried out hierarchical clustering (Fig 2). This analysis highlights that the four samples belonging to one of the six individuals cluster together in most cases. By contrast we did not find comparable clustering for any of the four tested blood sampling conditions.

### ANOVA of most variable miRNAs

**Fig 3:**
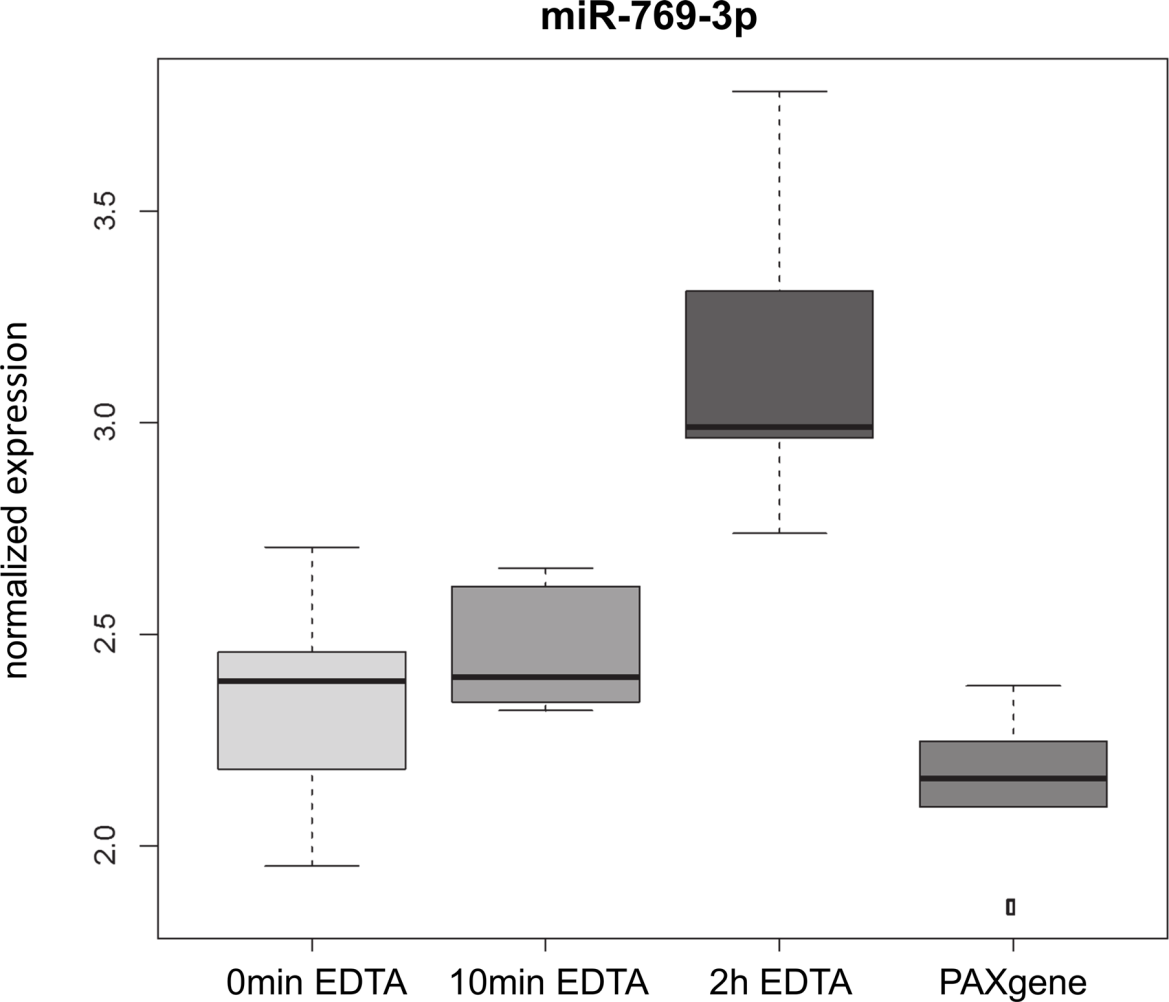
Box-plots of normalized abundance values of miR-769-3p for the 6 individuals under the four blood sampling conditions. The most obvious change of abundance of miR-769-3p was found in blood that was transferred in PAXgene^TM^ blood RNA tubes 2 h after phlebotomy.

**Fig 4:**
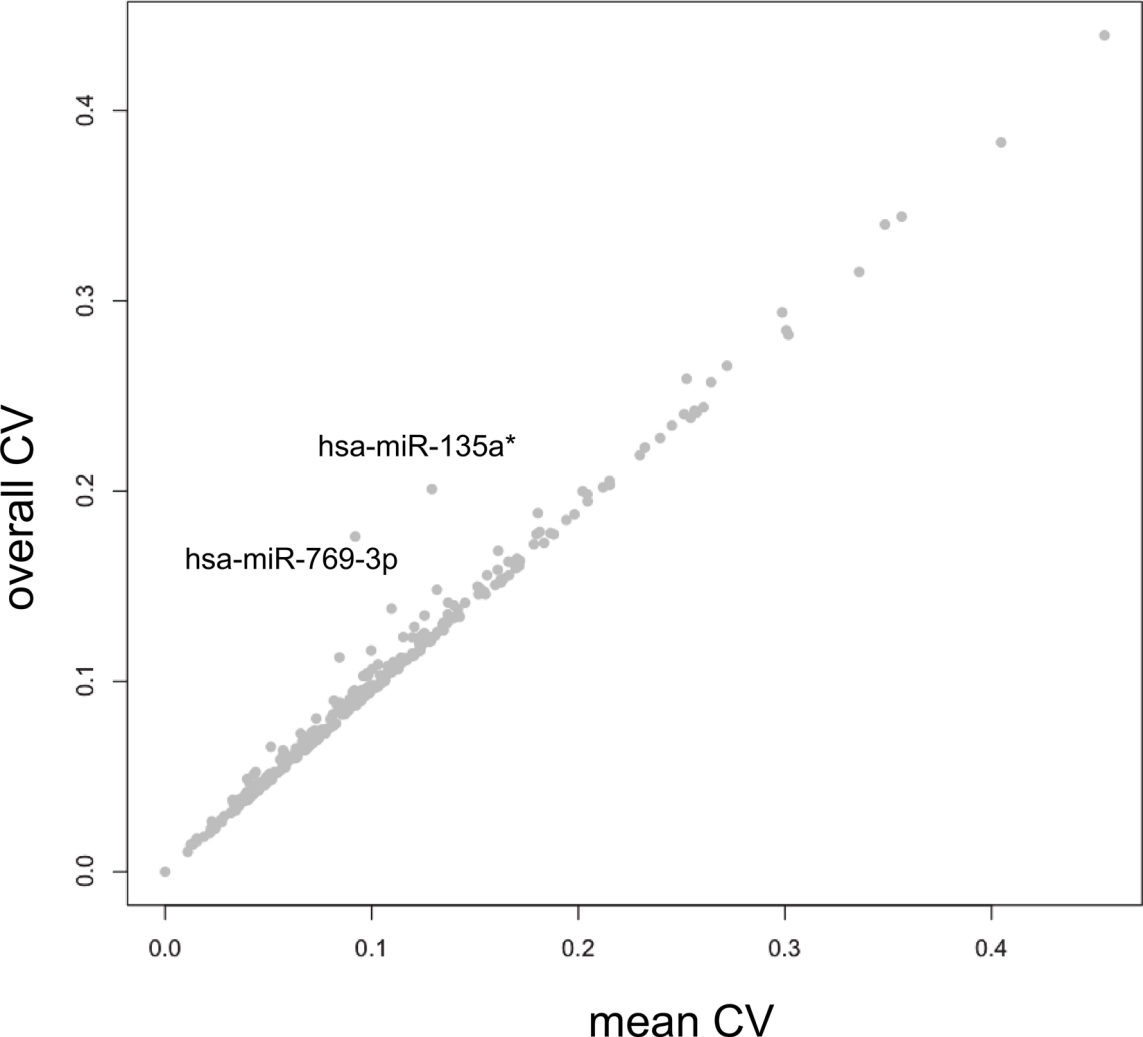
Scatter plot of the mean coefficient of variation (CV) for the four blood sampling conditions versus the overall CV. Both miR-769-3p and miR-135a* showed a higher overall CV as compared to their average CV within each of the four conditions indicating a condition specific abundance of both miRNAs.

Based on the 321 miRNAs that were expressed in at least 25% of the samples, we determined miRNAs that show an altered abundance in at least one of the four conditions. By performing an analysis of variance (ANOVA) we identified miR-769-3p with an adjusted p-value of 0.003. Notable, miR-769-3p was also identified by the above analysis of the binary data. Fig 3 shows the comparison of the normalized expression of the miR-769-3p for the 6 individuals under the four blood sampling conditions. To further define the influence of the different blood sampling conditions we calculated the coefficient of variation (CV), i.e. the quotient of standard deviation and mean value. This CV was computed for all 321 miRNAs and for each of the four conditions. While the minimal average CV was observed for 0min EDTA samples (9.4%) highest CV was observed for PAXgene samples (10.7%). Although the difference was small, it was statistically significant (two tailed paired t-test p-value of 0.0045). This fact can be due to at least two factors, either slightly increased technical variations in PAXgene samples or an improved detection of inter-individual deviations. To discover miRNAs that have a low in-group variation but large overall variation, we calculated the mean CV value across the four conditions as compared to the overall CV across all 24 samples. This analysis identified miR-769-3p and miR-135a* both of which showed a higher overall CV as compared to their average CV within each of the four conditions. In the scatter plot in Fig 4 both miRNAs are shown as clear outliers. While these results indicate a condition specific abundance for both miRNAs they also demonstrate again that the majority of the tested miRNAs does not show an abundance that differs under the four different blood sample conditions.

Since the same 6 individuals were profiled under the four blood sample conditions, our approach allowed a paired analysis between the different conditions (Table 1). In detail, we calculated for all possible six pair-wise comparisons (EDTA 0min versus EDTA 10min, EDTA 0min vs. EDTA 2h, EDTA 0min vs. PAXgene, EDTA 10 min vs. EDTA 2h, EDTA 10min vs. PAXgene, and EDTA 2h vs. PAXgene) paired t-tests for each miRNA. At an alpha level of 0.05, we found 44 miRNAs of the 321 miRNAs significant for the comparison EDTA 10min vs. EDTA 2h (13.7%), and 41 miRNAs significant for the comparison between EDTA 2h vs. PAXgene (12.8%). Only 14 miRNAs were significant for the comparisons between EDTA 0min vs. 10min (4.4%). After adjustment for multiple testing only four miRNAs including miR-769-3p remained significant for the comparison between EDTA 2h and PAXgene. The microarray based expression values of these 4 miRNAs were validated by qRT-PCR (S1 Fig). For hsa-miR-769-3p the microarray-based p-value was 0.00013, in qRT-PCR replicates we observed the same significant up-regulation after 2h EDTA storage (p = 0.004). Importantly, the difference between PAXgene and EDTA 10min was also significant in the qRT-PCR results (p = 0.03). This matched well with the microarray results prior to adjustment for multiple testing. For hsa-miR-1915 and hsa-miR-4275, the same trends were observed, however, missing the alpha level of 0.05. In case of hsa-miR-4270 we did not succeed to discover concordant patterns between qRT-PCR and microarrays.

**Table 1:** Paired analysis between the different conditions. For all possible six pair-wise comparisons paired t-tests for each miRNA was computed. The table shows the results for four miRNAs that remained significant for the comparison between EDTA 2h and PAXgene at an alpha level of 0.05, after adjustment for multiple testing.

	0min versus 10min	0min versus 2h	10min versus 2h	0min versus PAXgene	10min versus PAXgene	2h versus PAXgene
**# significant (raw)**	14	15	44	33	22	41
**# significant (adj)**	0	0	0	0	0	4
hsa-miR-769-3p	0.23281	*0*.*00024*	*0*.*00106*	*0*.*00770*	*0*.*00078*	*0*.*00013*
hsa-miR-4257	0.54596	0.18843	0.39395	0.93373	0.75646	*0*.*00013*
hsa-miR-4270	0.43674	0.57361	*0*.*00028*	*0*.*04290*	*0*.*00130*	*0*.*00037*
hsa-miR-1915	0.72441	0.52179	0.11235	0.24386	0.12608	*0*.*00054*

*Italic* = p-value <0.05 (before or after adjustment).

In conclusion, our results suggest that the duration of the EDTA influence has an impact on the miRNA abundances with more changes between 10 min and 2 hours as compared to the changes between 0 min and 10 min.

## Discussion

In the present study we analyzed the influence of EDTA as an anticoagulant on the miRNA expression pattern in peripheral blood specimen collected form healthy individuals. The blood was stored for different time periods in the blood collection tube containing EDTA and then transferred into PAXgene RNA blood tubes that contain a reagent to directly lyse blood cells and stabilize their RNA content. These samples were compared with blood specimen directly collected in PAXgene tubes. On average 283 miRNA were detected per sample, while 201 of all analyzed miRNA were detected in all 24 tested samples and 806 miRNAs were not detected in any of the tested samples. In general, we found a rather homogenous pattern of the miRNA abundance among the 24 samples that were collected from the six individuals each under four different blood sample conditions. Our analysis also indicated a higher similarity between miRNA patterns that stem from one of the six individuals than between the miRNA patterns that stem from the same blood sample condition.

As for the miRNAs that showed an altered abundance under specific conditions, we identified hsa-miR-769-3p that was not detected in any of the six PAXgene blood samples, but in all EDTA 2h samples. Hsa-miR-769-3p was also the only miRNA that showed a significantly different abundance between the 4 blood sample conditions by an ANOVA analysis (adj. p-value 0.003). Hsa-miR-769-3p was previously reported to be highly expressed in platelets [19]. In our study the significant abundance of this miRNA under EDTA conditions may be due pseudothrombozytopenia, which is an *in vitro* effect that results in the formation of platelet aggregates under the influence of EDTA [20]. Although platelets do not harbor a nucleus, they contain up to 32% of all human genes on the mRNA level [21], possess essential components of the translational machinery, and can respond to external stimuli [21]. Recently, Nagalla et al. reported expression of 284 miRNAs in platelets. These miRNAs were differentially expressed according to platelet aggregation with epinephrine [22]. In this study only hsa-miR-769-5p but not hsa-miR-769-3p was detected in all analyzed samples. As mentioned before, other studies found hsa-miR-769-3p expressed in platelets [19]. However, Nagalla et al. do not clearly state how they define detection and non-detection of a miRNA [22].

Besides hsa-miR-769-3p, the expression of hsa-let-7e seems also to be induced due to the 2h storage in EDTA tubes. Currently, there is, however, no other study investigating the expression of hsa-let-7e in blood. An additional miRNA that seems to be affected by anticoagulant EDTA was hsa-miR-135a*. Although this miRNA was detected in 18 of 24 tested samples it showed a higher overall CV among all 24 samples as compared to average CV for the four conditions. Other studies show that hsa-miR-135a* is expressed in peripheral blood lymphocytes and seems to be involved in immune/inflammatory response [23].

## Conclusion

It is known that the blood proteome or the transcriptome is ex vivo altered due to exposure to EDTA as an anticoagulant. We demonstrate how the blood miRNA pattern is affected EDTA. We show that the miRNA patterns are altered by short storage in EDTA but even stronger by prolonged storage in EDTA. We identified specific miRNAs namely miR-769-3p, of which the abundance is strongly altered by the different blood sampling conditions. Such altered miRNAs require specific attention in future blood-based miRNA biomarker studies. Overall, however, we found a rather homogenous pattern of the miRNA abundance. We found a stronger similarity among the miRNA pattern that belongs to a specific donor. No comparable clustering was, however, found for any of the four tested blood sampling conditions. Our study helps to better understand the impact of blood collection systems on the miRNA abundance, knowledge, which in turn is important for all studies that measure miRNAs in blood as clinical biomarkers.

## Supporting Information

S1 Table List of detected miRNAs in the 24 experiments. (XLSX)

S1 Fig Comparison of the microarray data (y-axis left = expression value in log scale) and the qRT-PCR results (y-axis left = ΔCt value). The x-axis indicates the 4 blood samples.
(JPG)
