# Supplementary figures and images for: Towards Clinical Applications of Blood-Borne miRNA Signatures: The Influence of the Anticoagulant EDTA on miRNA Abundance
Source: PLoS One. 2015 Nov 23;10(11):e0143321. doi: 10.1371/journal.pone.0143321 (PMC4658123; doi:10.1371/journal.pone.0143321)

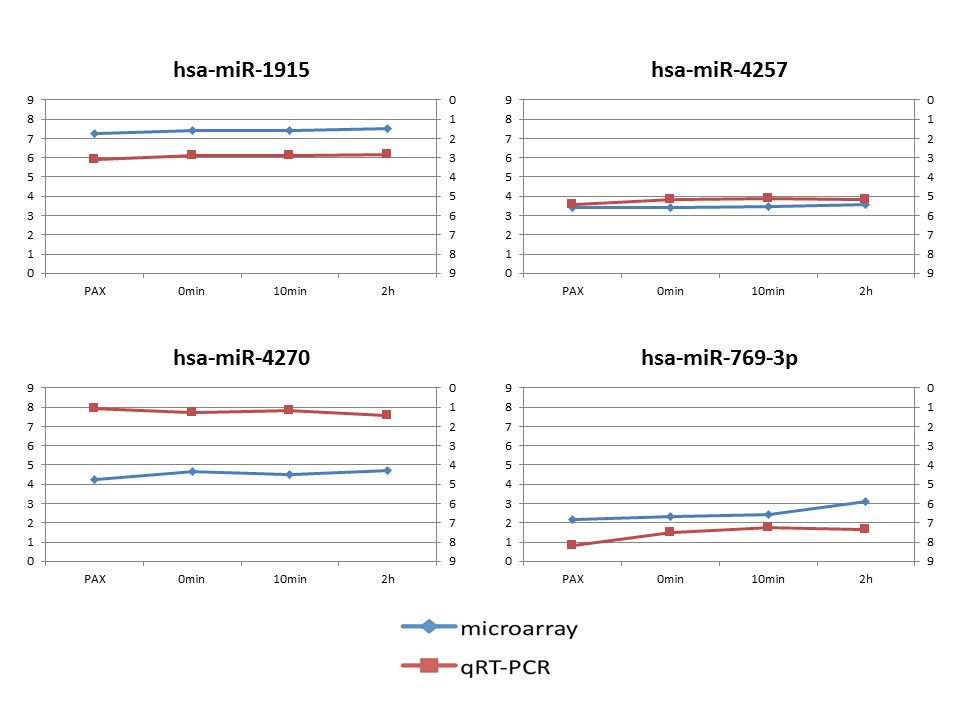

Supplement: S1 Fig — The x-axis indicates the 4 blood samples. (JPG) [file pone.0143321.s002.jpg]
